# Geographic atrophy phenotype identification by cluster analysis

**DOI:** 10.1136/bjophthalmol-2017-310268

**Published:** 2017-07-20

**Authors:** Jordi Monés, Marc Biarnés

**Affiliations:** 1 Institut de la Màcula, Barcelona, Spain; 2 Barcelona Macula Foundation, Barcelona, Spain

**Keywords:** age-related macular degeneration, geographic atrophy, soft drusen, reticular pseudodrusen, cluster analysis

## Abstract

**Background/aims:**

To identify ocular phenotypes in patients with geographic atrophy secondary to age-related macular degeneration (GA) using a data-driven cluster analysis.

**Methods:**

This was a retrospective analysis of data from a prospective, natural history study of patients with GA who were followed for ≥6 months. Cluster analysis was used to identify subgroups within the population based on the presence of several phenotypic features: soft drusen, reticular pseudodrusen (RPD), primary foveal atrophy, increased fundus autofluorescence (FAF), greyish FAF appearance and subfoveal choroidal thickness (SFCT). A comparison of features between the subgroups was conducted, and a qualitative description of the new phenotypes was proposed. The atrophy growth rate between phenotypes was then compared.

**Results:**

Data were analysed from 77 eyes of 77 patients with GA. Cluster analysis identified three groups: phenotype 1 was characterised by high soft drusen load, foveal atrophy and slow growth; phenotype 3 showed high RPD load, extrafoveal and greyish FAF appearance and thin SFCT; the characteristics of phenotype 2 were midway between phenotypes 1 and 3. Phenotypes differed in all measured features (p≤0.013), with decreases in the presence of soft drusen, foveal atrophy and SFCT seen from phenotypes 1 to 3 and corresponding increases in high RPD load, high FAF and greyish FAF appearance. Atrophy growth rate differed between phenotypes 1, 2 and 3 (0.63, 1.91 and 1.73 mm^2^/year, respectively, p=0.0005).

**Conclusion:**

Cluster analysis identified three distinct phenotypes in GA. One of them showed a particularly slow growth pattern.

## Introduction

Geographic atrophy (GA) is characterised by progressive loss of retinal pigment epithelium (RPE), adjacent photoreceptors and choriocapillaris.^1^ Therefore, areas affected by GA demonstrate an absolute scotoma.^2^ GA is responsible for a third of the cases of advanced age-related macular degeneration (AMD)^3^ and may affect a quarter of patients older than 90 years.^4^ There is no effective therapy to prevent, slow or revert the progression of GA. Indeed, GA progresses relentlessly with a remarkable interindividual variation.^5^ In the GAIN study,^6^ GA growth ranged from 0.11 to 5.55 mm^2^/year. The identification of potential GA phenotypes related to fast or slow progression is, therefore, important for several reasons: it would enable an individualised prognosis, define optimal eligibility for clinical trials, allow the phenotype–genotype correlation to be refined and provide a better understanding of disease pathogenesis.

There have been approaches to identify GA phenotypes in the past, and the use of fundus autofluorescence (FAF) imaging in the FAM study^7^ is perhaps the best example. In FAM, eyes were classified according to 10 different categories (phenotypes) based on the distribution of increased autofluorescence around areas of atrophy, which is heterogeneous, as recently reported by Gliem *et al*.^8^ This resulted in the discovery of an association between FAF patterns and growth rate. This classification is clinically useful, mainly for extreme cases, but was developed subjectively and showed low interobserver agreement.^9^ In addition, FAF phenotypes have been recently associated with baseline area of atrophy,^6^ suggesting they may reflect different stages of the disease rather than a true phenotype.^10^ Different fundus features in patients with GA may be present together, representing a particular phenotype that may have prognostic value (ie, a faster or slower growth rate). In addition to FAF characteristics, the fundus of patients with GA can be described according to drusen type (soft or reticular pseudodrusen (RPD)) and its extent, foveal atrophy, greyish appearance on FAF and subfoveal choroidal thickness (SFCT). The process of phenotype classification, however, is entirely subjective and may be prone to low interobserver reproducibility and/or bias. Several statistical tools such as latent class analysis or cluster analysis can be used to provide a more objective classification. Cluster analysis is a data-driven, statistical approach that groups observations into clusters when group membership is not previously known such that members of a cluster are more similar to each other than to members in other cluster groups.^11^

The aim of this study was to explore if cluster analysis could identify subgroups of patients with GA defined according to their fundus features.

## Material and methods

### Patient selection and setting

GAIN (NCT01694095) was a prospective, observational study that aimed to determine the risk factors associated with progression of GA secondary to AMD. It was conducted from 2009 to 2013 at the Institut de la Màcula (Barcelona, Spain) and has been described elsewhere.^6^ The information and images collected during GAIN were reviewed retrospectively for the purposes of the present study.

Patients were enrolled in the GAIN study if they met the following inclusion criteria: men or women ≥50 years with GA secondary to AMD, a minimum area of atrophy of 0.5 disk areas (1.27 mm^2^) on 35° colour fundus photography (CFP, using the TRC 50DX IA camera, IMAGEnet; Topcon, Tokyo, Japan) and ≥6 months of follow-up. Patients were excluded if the RPE atrophy was deemed to be secondary to other causes such as macular dystrophy or high myopia; there was a previous history of neovascular AMD or other significant maculopathy; GA contact with peripapillar areas of atrophy; inability to measure the whole area of atrophy; previous history of laser in the macula, antiangiogenic therapy or ocular surgery (aside from phacoemulsification) or insufficient imaging quality. Only one eye from each patient was included; in bilateral cases, the study eye was randomly selected.

### Procedures

As part of the GAIN protocol, all patients underwent a complete ophthalmic assessment after pupil dilatation with 1% tropicamide and 10% phenylephrine. Imaging included non-stereoscopic 35° CFP, 30° infrared (IR; λ=820 nm) and FAF (excitation λ=480 nm, emission λ ~500–700 nm) with the Spectralis HRA+OCT (Heidelberg Engineering, Heidelberg, Germany) in the high-resolution mode (1536×1536 pixels) with a minimum automatic real time (averaging) of 10 images. Spectral domain optical coherence tomography (SD OCT) with the same instrument was used to acquire a horizontal, high-resolution, 30° B-scan with an averaging ≥20 B-scans. Fluorescein angiography was performed only if required to rule out concomitant disorders. All images were acquired by certified photographers.

Features used to describe the fundus of patients with GA were presence of numerous soft drusen and RPD, presence of primary foveal atrophy, increased FAF, greyish FAF appearance and SFCT. A multimodal approach was used to identify and classify these features: (1) CFP was used to identify soft drusen,^12^ (2) IR and multimodal colour imaging improves the visualisation of outer retinal structures and was therefore used to determine RPD^13 14^; (3) FAF was used to detect the area of atrophy and its appearance, such as greyish (which has been associated with faster growth)^15^ or black hue. This also shows the topographical distribution of lipofuscin (from long-lasting residual bodies within the RPE) as areas of increased autofluorescence and hence indicates the extent of RPE abnormalities in the areas adjacent to the atrophy, although other causes of increased FAF are also possible and (4) SD OCT allows the high-resolution, cross-sectional visualisation of outer retinal anatomy and the determination of choroidal thickness.^16^

**Figure 1:**
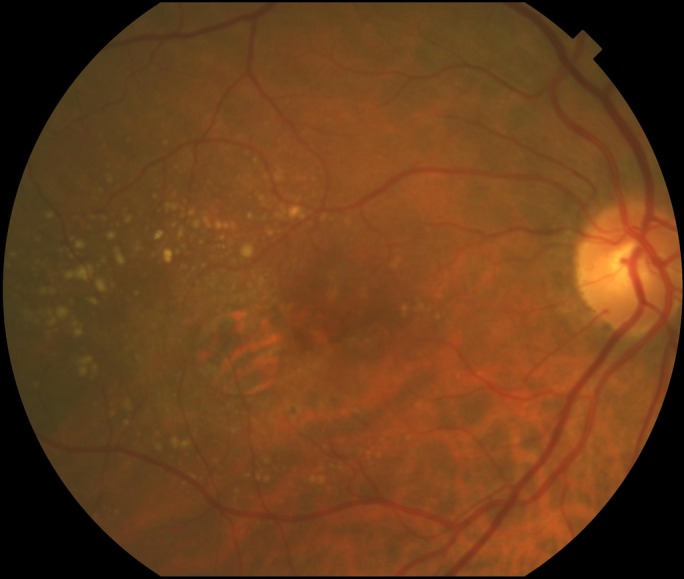
Reference for establishing soft drusen load. A quantity of soft drusen larger than or equal to that shown in this figure was considered as a fundus with a high soft drusen load.

Figure 1 shows the minimum quantity of soft drusen from which a patient was considered to have a high drusen load. High RPD load were defined as small hyporeflective oval or circular lesions surrounded by an isoreflective or mildly hyper-reflective annulus on IR or multimodal colour imaging and/or subretinal hyper-reflective material elevating or interrupting the ellipsoid zone^17^ on SD OCT in ≥25% of B-scans. For soft drusen and RPD, the classification was either yes or no according to the aforementioned criteria. Primary foveal atrophy was considered present if there was loss of pigmentation in an area that included the geometric centre of the foveal avascular zone on CFP or IR. Increased FAF corresponded to ‘banded’ or ‘diffused’ patterns as defined in the FAM study^7^; patients categorised as ‘none’ and ‘focal’ were considered to have no or minimal increased FAF, whereas patients with an undetermined pattern were excluded. The scale of grey of atrophy was subjectively determined on FAF and classified as greyish FAF appearance or black. SFCT was measured from the external border of the RPE to the uveoscleral junction just under the foveola using the calliper function of the SD OCT. All classifications were made by a senior retinal specialist (JM) except for FAF patterns, in which two experienced observers (Fabio Trindade and MB) independently determined the FAF pattern. In cases of disagreement, a senior observer (JM) arbitrated. A consensus was reached in all cases.

The area of atrophy was measured on FAF by a single observer (MB) using the Region Finder software, version 2.4.3.0 (Heidelberg Engineering), with good intra-observer agreement (see GAIN study).^6^ Rate of growth was determined by subtracting the area of atrophy (in square millimetre) at the last visit from area of atrophy at baseline divided by time between visits (in years). Atrophy growth was measured as increase in surface area (square millimetre per year) and linear growth (millimetre per year) using the square root transformation of each measure.^18^

### Statistical analysis

Univariate statistics were used to describe the complete sample, using medians (IQR) for quantitative variables and percentages for categorical variables. The formation of groups in cluster analysis is based on the proximity of the characteristics that define each observation in the multivariate space, that is, on the presence of fundus features that have a tendency to be present together in different patients. Therefore, the analysis consisted of two main steps: first, cluster analysis was used to create the groups; second, fundus features were compared between groups and each phenotype characterised. Missing values were estimated using simple imputation.^19^

An agglomerative approach was used for the cluster analysis, which starts with all eyes as independent observations and proceeds by grouping them until a single cluster that includes all eyes is formed. A hierarchical method (in which the number of final clusters was not predefined) was used. The Calinski-Harabasz pseudo-F index was used to determine the optimal number of groups (phenotypes).^20^ All quantitative variables were standardised (rescaled to a mean value of 0 and an SD of 1).

Between-phenotype comparisons for fundus features including growth were conducted using the Kruskal-Wallis test or the exact Fisher’s test. Comparisons of growth rate (in square millimetre per year and millimetre per year) between pairs of phenotypes were made using the Mann-Whitney test. Three linear regression models were fitted to determine the impact of baseline area of atrophy (an important predictor of growth), the phenotype or both on growth rate. Statistical analyses were conducted using Stata IC 13.1 (Stata, Texas, USA). A two-sided p value of <0.05 was considered statistically significant.

## Results

The study included data from 77 out of the original 82 patients enrolled in GAIN (data from four patients were excluded because their FAF pattern was ‘undetermined’ and one patient was excluded because he consistently formed a single cluster). Mean follow-up was 21.6 months (range 6 to 41 months). Median age was 79 years (IQR 11, range 52 to 97 years old); 70.1% were female (54/77), and all were Caucasian. The median baseline area of atrophy was 5.47 mm^2^ (IQR 9.82, range 1.25 to 37.83 mm^2^). Median GA area growth rate was 1.56 mm^2^/year (IQR 1.48, range 0.11 to 5.55 mm^2^/year), whereas GA linear growth (using the square root transformation) was 0.32 mm/year (IQR 0.24, range 0.02 to 0.72 mm/year). All fundus features could be measured in all patients except for SFCT for two patients (2/77, 2.6%), and these values were imputed as stated previously.

**Figure 2:**
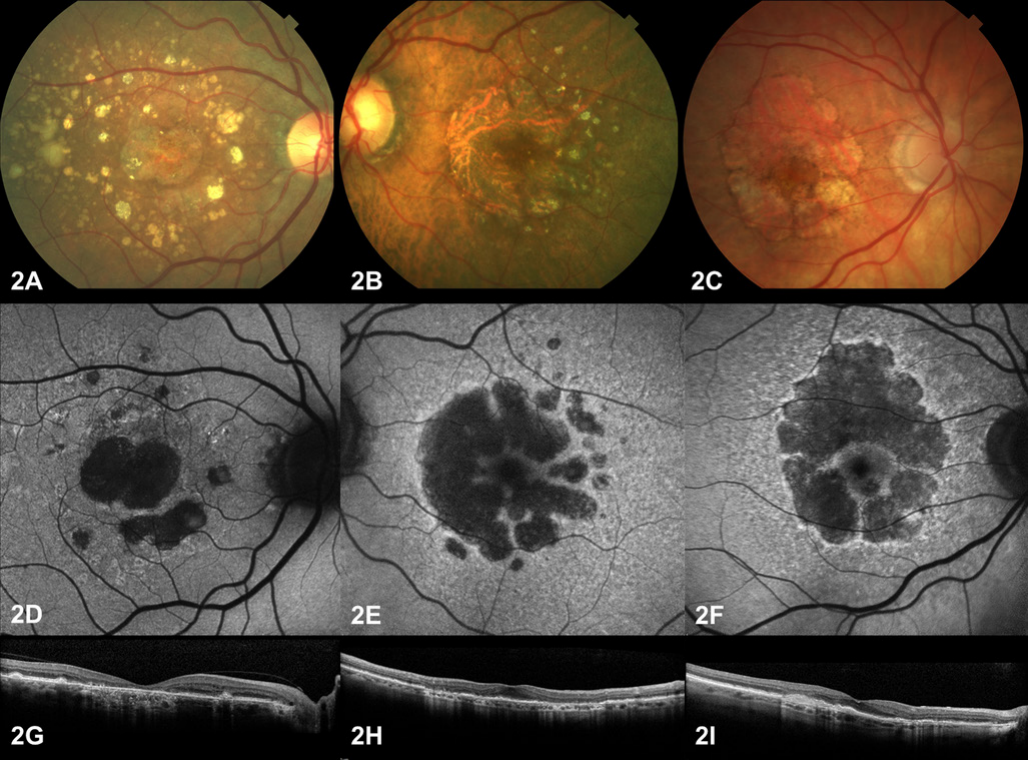
Examples of phenotypes. Representative fundus photographs (A–C), FAF (D–F) and spectral domain optical coherence tomography (G–I) images from phenotypes 1 (first column), 2 (second column) and 3 (third column). Phenotype 1, with slow growth, is characterised by foveal atrophy and a high soft drusen load, whereas phenotype 3 showed a high reticular pseudodrusen load, a perifoveal area of atrophy with increased FAF and a thin subfoveal choroidal thickness. Phenotype 2 presented intermediate features between phenotypes 1 and 3. FAF, fundus autofluorescence.

Cluster analysis identified three groups of patients based on fundus characteristics (table 1). From phenotypes 1 to 3, there was a progressive decrease in the prevalence of soft drusen load, foveal atrophy and SFCT. In contrast, the prevalence of high RPD load, high FAF and greyish FAF appearance increased from phenotypes 1 to 3. Figure 2 shows representative images for each cluster.

**Table 1:** Characteristics of the phenotypes from cluster analysis

	Phenotype			
Characteristics	1 (n=16, 20.8%)	2 (n=52, 67.5%)	3 (n=9, 11.7%)	p Value
Soft drusen	56.3 (29.9 to 80.2)	34.6 (22.0 to 49.1)	0.0 (0 to 33.6)	0.013
RPD	50.0 (24.7 to 75.3)	80.8 (67.5 to 90.4)	100 (66.4 to 100)	0.012
Foveal atrophy	100 (79.4 to 100)	0.00 (0 to 6.8)	0.00 (0 to 33.6)	<0.001
High FAF	12.5 (1.6 to 38.3)	73.1 (59.0 to 84.4)	88.9 (51.8 to 99.7)	<0.001
Greyish FAF appearance	0.0 (0 to 20.6)	0.0 (0 to 6.8)	100 (66.4 to 100)	<0.001
SFCT (µm)	197 (118 to 253)	134 (82 to 179)	51 (26 to 60)	0.0001
Area growth rate (mm^2^/year)	0.63 (0.32 to 1.32)	1.91 (1.27 to 2.59)	1.73 (1.24 to 2.06)	0.0005
Linear growth rate (mm/year)	0.17 (0.09 to 0.32)	0.33 (0.20 to 0.43)	0.32 (0.24 to 0.42)	0.022

Results are shown as percentage (95% CI) for categorical variables and median (25th and 75th percentiles) for quantitative variables. The p value corresponds to the comparison between the three groups.

FAF, fundus autofluorescence; RPD, high reticular pseudodrusen load; SFCT, subfoveal choroidal thickness.

When considering area of GA growth rate (square millimetre per year), the median growth was 0.63 (IQR 1.00), 1.91 (IQR 1.33) and 1.73 (IQR 0.82) mm^2^/year in phenotypes 1, 2 and 3, respectively (p=0.0005). A paired comparison showed growth was slower in phenotype 1 compared with phenotype 2 (p=0.0002) or 3 (p=0.0055), but there were no differences between phenotypes 2 and 3 (p=0.58). Similar results were found for linear GA growth rate using the square root transformation (millimetre per year): median growth was 0.17 (IQR 0.23), 0.33 (IQR 0.23) and 0.32 (IQR 0.18) mm/year in phenotypes 1, 2 and 3, respectively (p=0.022). Linear growth was smaller in phenotype 1 compared with phenotype 2 (p=0.01) or 3 (p=0.02), but it was similar between phenotypes 2 and 3 (p=0.78).

Median age did not differ between groups and was 76 (IQR 10), 79 (IQR 10.5) and 85 (IQR 6) years in phenotypes 1, 2 and 3, respectively (p=0.13). The baseline area of atrophy was different in phenotypes 1, 2 and 3 (3.20 (IQR 2.94), 7.75 (IQR 11.13) and 5.68 (IQR 5.74) mm,^2^ respectively, p value=0.0026), but as previously shown, differences in growth between phenotypes remained statistically significant when using the square root transformation, suggesting that baseline area of atrophy was not the cause of the different growth rate between groups. In addition, linear regression showed an association between baseline area of atrophy and GA growth rate in square millimetre per year (p=0.003). This was also the case for the ocular phenotype as a categorical variable (p=0.001). When both variables were simultaneously included in a multiple linear regression model, baseline area of atrophy had borderline statistical significance (p=0.05) and ocular phenotype was statistically significant (p=0.01).

## Discussion

Considerable efforts have been made in the last two decades to identify different phenotypes in neovascular AMD^21–24^ and the response of these phenotypes to emerging therapies for choroidal neovascularisation.^25 26^ Recently, GA has gained renewed interest and new therapies to prevent or halt the rate of atrophy growth have emerged. To date, these clinical trials have been unsuccessful, and this has driven the scientific community to search for subgroups of patients with different prognosis in terms of disease progression and, possibly, related but different disease pathogenesis. By identifying different phenotypes through the use of biomarkers, it may be possible to correlate genotype with progression and investigate potential different pathways within the atrophic form of AMD. Unfortunately, besides FAF patterns with its inherent limitations, no other biomarkers have been used to stratify patients in clinical trials for GA. Since current investigational therapies are unlikely to result in a huge therapeutic benefit across the range of patients with GA, identifying different prognostic phenotypes may help distinguish subgroups with subtle or limited therapeutic responses and hence allow use of these as eligibility criteria and/or stratification factors in future trials.

Our main findings suggest that there are at least three phenotypes in patients with GA based on non-invasive multimodal imaging and a data-driven approach, cluster analysis. Growth rate was slower in one of the phenotypes as compared with the other two and may thus represent a subgroup with relatively good prognosis in terms of atrophy enlargement. This group was characterised by a high soft drusen load, underscoring the differential influence that drusen type may confer on growth rate.

The three phenotypes differed in all measured characteristics (p≤0.013). Phenotype 1 (n=16, 20.8% of eyes) was characterised by foveal atrophy in all cases, a high soft drusen load, thick choroid and usually no or small increased FAF. These patients showed the slowest growth rate (0.63 mm^2^/year). Phenotype 3 (n=9, 11.7%) was the least common; all cases had high RPD load, a greyish FAF appearance and a thin choroid. These eyes showed a moderate growth rate (1.73 mm^2^/year). Phenotype 2 was the most common (n=52, 67.5% of eyes) and showed intermediate features between phenotypes 1 and 3, and only rarely showed foveal atrophy or a greyish FAF appearance (0%, 95% CI 0% to 6.8%). A perifoveal area of atrophy, presence of RPD, thin choroid and increased FAF have been previously associated with faster progression,^6 7 15 27^ but these features are not reported to present together in any given eye.

Although it may appear that there is a continuum from phenotypes 1 to 3 in terms of certain fundus features and even patient age (which may imply that this represents different stages in the disease), this may not be the case. Most patients in phenotype 1 are expected to have foveal atrophy (100%, 95% CI 79.4 to 100), which is rarely seen in patients with phenotype 2 (0%, 95% CI 0% to 6.8%) or 3 (0%, 95% CI 0% to 33.6%). This argues against a ‘different stages’ hypothesis and adds further support to the argument that these groups may indeed represent true different phenotypes.

These phenotypes were associated with growth rate independently of baseline area of atrophy (a strong predictor of growth), as shown by the use of the square root transformation and by linear regression. Therefore, these phenotypes could be used to provide a prognosis at the time of diagnosis and identify patients with a slow growing form of GA (phenotype 1). This has implications for risk stratification, for defining optimum eligibility in clinical trials and for improving study efficiency. Furthermore, exclusion of patients with slow progressing GA from clinical trials is useful as it avoids exposing patients with good prognosis to potentially toxic treatments and minimises the time required to show a beneficial effect of the experimental intervention. In addition, most single nucleotide polymorphisms discovered so far are associated with both forms of late AMD^28^ despite the obvious fundus heterogeneity between the dry and the neovascular forms of the disease. This calls for refined phenotyping to help elucidate phenotype–genotype correlations and clarify the role of genetics and the relative contribution of fundus features (RPD, soft drusen, thin choroid, etc) on disease pathogenesis. Finally, these phenotypes may help to define specific cellular pathways and suggest roles for specific features, such as type of drusen on the dynamics of GA progression. One of the most intriguing findings of this study is that soft drusen-related GA, which frequently starts at the fovea, has a significant slower rate of progression compared with GA related mainly to high RPD load, which tends to preserve the fovea in the early stages.

The main strength of this study is the objective identification of a phenotype with specific characteristics and slow progression. The GA phenotype related to abundant soft drusen shows early atrophy of the centre of the fovea with early impairment of visual acuity but has a slow progression that allows a relatively good visual function over the long term. These patients should be stratified separately in small clinical trials addressing GA. On the other hand, this study has several limitations. First, it cannot be stated that the differences between fundus features are the cause of the different growth rates because other factors not included in the analyses may be responsible for this, such as refractile drusen^29^ or other hitherto unknown factors. Second, the choice of categorisation of some of the variables (soft drusen load, RPD, etc) was arbitrary; as new evidence emerges, our classification of fundus features may need to be changed accordingly, which may have an impact on the current phenotypic classification. Finally, future studies with larger cohorts should include exploratory and confirmatory data sets. The results are based on a method used previously in AMD^30^ and in other areas of ophthalmology with good results.^31 32^ The EYE-RISK study is assembling a large database of patients with AMD in Europe, which will be used to confirm these findings.

In summary, three phenotypes in GA were identified using cluster analysis, and at least one of them showed a slow growth pattern. This may be useful for determining prognosis and clinical trial eligibility as well as phenotype–genotype correlation.
